# New alternative to antidotes for novel oral anticoagulants and ticagrelor in the case of severe bleeding

**DOI:** 10.1186/s13054-020-2760-7

**Published:** 2020-02-11

**Authors:** Patrick M. Honore, Christina David, Rachid Attou, Sebastien Redant, Andrea Gallerani, David De Bels

**Affiliations:** ICU Department, Centre Hospitalier Universitaire Brugmann-Brugmann University Hospital, Place Van Gehuchtenplein, 4, 1020 Brussels, Belgium

Kuramatsu et al. reviewed current therapies for reversal of new oral anticoagulants (NOACs) and anti-platelet agents in patients with acute intracerebral hemorrhage [1]. In their comments on “Unspecific reversal approaches,” we believe that the authors have overlooked another new way to reverse this anticoagulation, especially with NOACs such as rivaroxaban and dabigatran and the new antiplatelet drug, ticagrelor [2]. In the case of severe intoxication with these NOACs or new anti-platelet agents, there is a promising new therapy based upon the use of the CytoSorb device [2]. CytoSorb can very efficiently remove NOACS and anti-platelets agents in order to restore normal coagulation and platelet function and to stop bleeding wherever it is occurring [2]. In their study, Angheloiu et al. were able to remove 99% of ticagrelor from human blood in less than 4 h when using CytoSorb [2]. They concluded that CytoSorb can remove representative molecules from two classes of agents—antiplatelet and anticoagulant—and in the future could complement the use of a newly developed specific monoclonal antibody reversal agent for ticagrelor, which is still in the pre-clinical phase and not yet available at the bedside [3]. In other experimental work by Koertge et al. [4], it was found that more than 91% of rivaroxaban could be removed from the blood during 1 h of use of CytoSorb [4]. This new therapy could perhaps complement the use of the antidote andexanet alfa [1], particularly if the antidote is not immediately available [4]. Lastly, Hassan et al. reported the intra-operative use of CytoSorb adsorption of ticagrelor and rivaroxaban in emergency open-heart surgery [5]. They concluded that this strategy is a safe and effective method to reduce bleeding complications induced by ticagrelor and rivaroxaban in that setting [5]. Studies comparing the two strategies (sorbents versus monoclonal antibodies) are urgently needed.

## Data Availability

Not applicable.

## Abbreviation

NOACs: New oral anticoagulants
